# Characterization of cerebral blood flow during open cardiac massage in swine: Effect of volume status

**DOI:** 10.3389/fphys.2022.988833

**Published:** 2022-10-04

**Authors:** Neerav Patel, Joseph Edwards, Hossam Abdou, David P. Stonko, Rebecca N. Treffalls, Noha N. Elansary, Thomas Ptak, Jonathan J. Morrison

**Affiliations:** ^1^ R. Adams Cowley Shock Trauma Center, University of Maryland, Baltimore, MD, United States; ^2^ Department of Surgery, Johns Hopkins Hospital, Baltimore, MD, United States

**Keywords:** resuscitation, hemorrhage, cardiopulmonary resuscitation, CPR, open cardiac massage, hypoperfusion, brain perfusion, coronary perfusion

## Abstract

**Introduction:** Patients in cardiac arrest treated with resuscitative thoracotomy and open cardiac massage (OCM) have high rates of mortality with poor neurological outcomes. The aim of this study is to quantitate cerebral perfusion during OCM using computed tomography perfusion (CTP) imaging in a swine model of normo- and hypovolemia.

**Methods:** Anesthetized swine underwent instrumentation with right atrial and aortic pressure catheters. A catheter placed in the ascending aorta was used to administer iodinated contrast and CTP imaging acquired. Cerebral blood flow (CBF; ml/100 g of brain) and time to peak (TTP; s) were measured. Animals were then euthanized by exsanguination (hypovolemic group) or potassium chloride injection (normovolemic group) and subjected to a clamshell thoracotomy, aortic cross clamping, OCM, and repeated CTP. Data pertaining to peak coronary perfusion pressure (pCoPP; mmHg) were collected and % CoPP > 15 mmHg (% CoPP; s) calculated post hoc.

**Results:** Normovolemic animals (*n* = 5) achieved superior pCoPP compared to the hypovolemic animals (*n* = 5) pCoPP (39.3 vs. 12.3, *p* < 0.001) and % CoPP (14.5 ± 1.9 vs. 30.9 ± 6.5, *p* < 0.001). CTP acquisition was successful and TTP elongated from spontaneous circulation, normovolemia to hypovolemia (5.7 vs. 10.8 vs. 14.8, *p* = 0.01). CBF during OCM was similar between hypovolemic and normovolemic groups (7.5 ± 8.1 vs. 4.9 ± 6.0, *p* = 0.73) which was significantly lower than baseline values (51.9 ± 12.1, *p* < 0.001).

**Conclusion:** OCM in normovolemia generates superior coronary hemodynamics compared to hypovolemia. Despite this, neither generates adequate CBF as measured by CTP, compared to baseline. To improve the rate of neurologically intact survivors, novel resuscitative techniques need to be investigated that specifically target cerebral perfusion as existing techniques are inadequate.

## Introduction

Open cardiac massage (OCM) and aortic cross clamping (x-clamping) is performed in trauma patients presenting in cardiac arrest *via* resuscitative thoracotomy (RT) as a last-ditch heroic maneuver. (Burlew et al., 2012; Morrison and Rasmussen, 2012; Morrison et al., 2013; Inaba et al., 2015; Carroll et al., 2020; DuBose et al., 2020; Madurska et al., 2021; Owattanapanich et al., 2021). The goal of OCM is to generate a coronary perfusion pressure sufficient to perfuse the myocardium and promote the return of a spontaneous circulation (ROSC). (Burlew et al., 2012; Morrison and Rasmussen, 2012; Morrison et al., 2013; Inaba et al., 2015; Carroll et al., 2020; DuBose et al., 2020; Madurska et al., 2021; Owattanapanich et al., 2021). X-clamping increases afterload and helps focus the limited cardiac output toward the heart and brain. A further benefit is that x-clamping will exclude any infra-diaphragmatic source of bleeding from the circulation.

Despite its attention in the literature, RT with OCM affords only 15% survivability from penetrating wounds and is as low as 1%–2% for blunt injury (Burlew et al., 2012; Inaba et al., 2015). For those rare patients who obtain ROSC, the neurologic sequela can be devastating. It is thought that only approximately 27% of patients will regain consciousness after 28 days, and only about half of patients who get ROSC will survive to hospital discharge (Loomba et al., 2015). Hypoxic-brain injuries from delayed ROSC often lead to poor neurological outcomes and are a significant contributor to morbidity and mortality (Kaye, 2005; Loomba et al., 2015). The goal of initial trauma resuscitation should be to rescue the cardiopulmonary system but while doing so, preserve and protect the central nervous system. Cerebral perfusion is an important resuscitation endpoint to assess the neurological status of survivors (van den Brule et al., 2018; Lorusso and Bělohlávek, 2020; Gil-Anton et al., 2020). Computed tomography perfusion (CTP) scanning offers a non-invasive method to assess for cerebral perfusion (Abdou et al., 2021; Abdou et al., 2022).

Cerebral perfusion during RT and OCM with x-clamping has not been well characterized using modern whole brain techniques which quantify cerebral blood flow (CBF). Furthermore, the effect of normovolemic versus hypovolemic states have also not been investigated. The aim of this study is to use CTP scanning in hypovolemic and normovolemic swine models to characterize brain perfusion and to correlate this with coronary hemodynamic metrics during RT with OCM.

## Materials and methods

### Study overview

Animal study protocols were approved by the University of Maryland, Baltimore Institutional Animal Care and Use Committee (IACUC) and conform to the National Institutes of Health guidelines for ethical animal research. The study used ten male Yorkshire swine (*Sus scrofa)* weighing between 30 and 60 kg. The animals were housed in individual cages next to each other in a temperature (21°C–23°C) and humidity (30%–70%) controlled environment. A timed diurnal light and dark cycle was used with light gradually introduced for 12 light h followed by light gradually reduced for 12 dark h. All animals were under veterinary supervision with species specific enrichment. The animal procurements were specifically scheduled to ensure animals were not kept in the vivarium for longer than 10 days. In the interest of preserving precious animal resources, this study enrolled animals used as controls for other IACUC approved study where animals used in device and drug testing were euthanized by either a controlled hemorrhage or a potassium chloride injection. None of the prior studies interfered with the heart or cerebrovasculature. The Animal Research: Reporting *In Vivo* Experiments (ARRIVE) 2.0 guidelines were followed for standardized reporting. (Percie du Sert et al., 2020).

**FIGURE 1:**
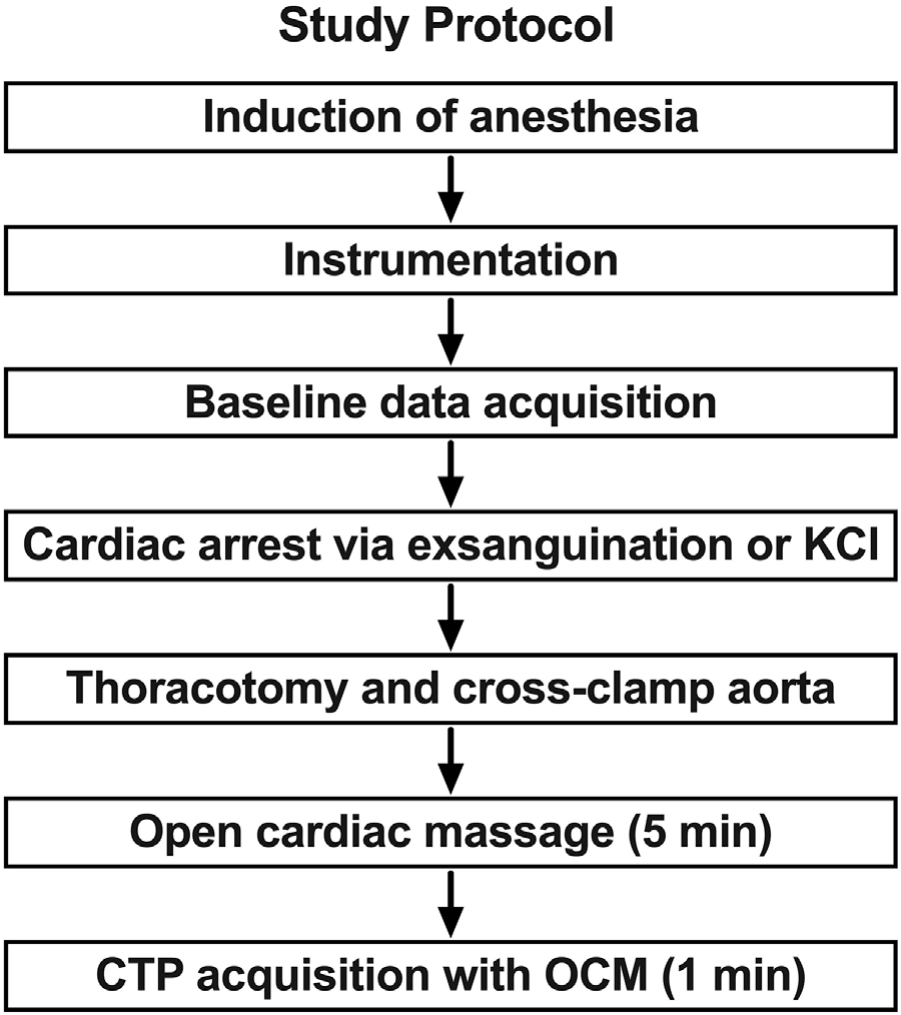
Study protocol and sequence. In phase 1 anesthesia is induced and the animal is prepared with catheter introduction and monitoring device placement. In phase 2 the animal is closely monitored, and baseline data is acquired. The animal is then exsanguinated to arrest or administer potassium chloride (KCl) in normovolemic model. This is followed by the placement of an aortic cross clamp in phase 3. Open cardiac massage (OCM) is provided while brain profusion is assessed with CT perfusion (CTP).

The study protocol consisted of three phases: instrumentation and baseline data acquisition, induction of arrest, and CPR (Figure 1). Animals in the hypovolemic group (*n* = 5) were induced into arrest by exsanguination and those in the normovolemic group (*n* = 5) were induced into arrest by potassium chloride injection.

### Animal instrumentation and monitoring

Subjects were sedated with Telazol (5 mg/kg)/Xylazine (2 mg/kg) *via* intramuscular injection. Animals were fasted for at least 12 h prior to the procedure to reduce the risk of regurgitation or aspiration (Muir et al., 2013; Grimm et al., 2015; Bradbury and Clutton, 2016). The chance of regurgitation in swine has been noted to be significantly decreased with water restriction for 6–12 h and food restriction for 12–24 h (Grimm et al., 2015). Using Isoflurane by facemask, general anesthesia was induced followed by orotracheal intubation. Animals were maintained under general anesthesia targeting a MAC of 1 throughout the experiment. The animals were placed on a warming blanket set to 37°C. The animals were mechanically ventilated using SIMV/PC mode with a fraction of inspired oxygen of 40% to maintain an end tidal pCO_2_ of 30–40 mmHg. Arterial blood gases were obtained as necessary to guide anesthesia and ventilation.

A 7 French (Fr) sheath was percutaneously introduced into bilateral brachial arteries to facilitate placement of a 5 Fr pigtail catheter and 5 Fr solid state pressure catheter in the ascending aorta using fluoroscopic guidance. The jugular vein was percutaneously accessed with a 7 Fr sheath to facilitate insertion of another solid-state pressure catheter into the right atrium to measure right atrial (RA) pressure. Contrast injection for CTP acquisition was given through the pigtail catheter *via* a brachial artery. A 14 Fr venous drainage cannula was placed in the left femoral vein to allow for controlled hemorrhage (van den Brule et al., 2018). Finally, a urinary cystostomy was performed for urinary drainage. The animals were monitored with electrocardiography (ECG), temperature probes, and real time arterial pressure tracings *via* the aforementioned vascular access sites. They were monitored for 1 h after instrumentation.

### Computed tomography perfusion technique

Our group has previously developed a CTP protocol using an OmniTom portable CT scanner (Neurologica, Danvers, MA) for large animal resuscitation research (Madurska et al., 2020; Madurska et al., 2021; Edwards et al., 2022a; Stonko et al., 2022). In brief, CTP is a technique which measures iodinated contrast transit through brain parenchyma. Contrast is given intra-arterially to increase resolving power, decrease volume of contrast required, and mitigate the effect of slow transit in hypovolemic subjects (Madurska et al., 2021; Stonko et al., 2022). Normovolemic CTP imaging was acquired to serve as a control. Intra-arterial contrast was given *via* pigtail catheter placed in the ascending aorta *via* the brachial artery at a rate of 2.5 ml/s for 3 s. The injection was initiated 5 s after the start of the CTP acquisition to allow brief capture of arterial inflow prior to contrast bolus arriving to the area of interest.

CT perfusion imaging is obtained from serial static axial acquisitions on a single CT slice immediately following contrast administration. After contrast administration, the density over time measured by contrast concentration in the cerebral tissue forms a time-concentration curve. For arterial blood flow reference, referred to as arterial input function (AIF), the internal carotid artery was used. The internal jugular vein was used for venous blood flow reference, referred to as venous output function (VOF). AIF and VOF curves were calculated in the MIStar program as the change in contrast concentration in cerebral tissue over time (i.e., the change in HU over time). The CTP analyzes the time-concentration curve in each voxel to approximate cerebral blood volume (CBV), CBF, and the mean transport time (MTT) of inflow-outflow. CBV is calculated based on the area under the AIF curve and measured as ml per 100 g of tissue (ml/100 g). CBF is calculated as the slope of the AIF and measured as 100 g of tissue per second (ml/100 g/min). MTT is the time in seconds of inflow and outflow within a portion of the tissue and can be calculated based on CBV/CBF.

### Normovolemic and hypovolemic groups

For the normovolemic arrest animals (*n* = 5), blood volume was maintained and potassium chloride was used for euthanasia. For the hypovolemic arrest animals (*n* = 5), a central venous cannula was connected to a peristaltic pump for controlled exsanguination. Blood was removed *via* the peristaltic pump at 100 ml/s *via* the venous cannula until cardiac arrest, where arrest was defined as systolic blood pressure (SBP) < 20 mmHg ting for >1 min. Once cardiac arrest was confirmed in both groups, a clam-shell thoracotomy was performed. The descending mid-thoracic aorta was dissected from its pleural attachments and an aortic cross-clamp was applied. The position of the pigtail catheter and pressure catheter were confirmed in the ascending aorta by manual palpation. The pericardial sac was incised in the cranio-caudal direction and the heart was delivered through the opening permitting cardiac massage.

### Cardiac massage

OCM was initiated at a rate of 100 compressions per minute by a surgical resident with at least 2 years of experience in OCM in human patients during RT. Adequate flow was defined by aortic pressures greater than 20 mmHg during compression as measured by the aortic pressure probe. 16. This method was repeated in all animals. CTP images were transferred to a Picture Archiving and Communication System (PACS) for storage and further analysis.

### Data capture and analysis

Pressure data was captured continuously from the RA and aortic root using data acquisition software (LabChart; ADInsturements, Sydney, Australia). This data was used to derive coronary perfusion pressure (CoPP) based on aortic to RA pressure gradient during diastole. To analyze CoPP data, consecutive 10 s intervals were extracted for each animal. This methodology of analyzing smaller segments during OCM has been validated in prior work. (Paradis et al., 1990; Edwards et al., 2022b). In the study by Paradis et al. (1990), CoPP > 15 mmHg was associated with higher likelihood of ROSC during OCM. As such, CoPP > 15 mmHg was measured in this study as a determinant of adequate CoPP for ROSC. The estimated maximum coronary flow was calculated using the functional flow reserve (FFR), equation: $FFR=\frac{Coronary\;Pressure-Venous\;Pressure}{Mean\;Aortic\;Pressure-Venous\;Pressure}$ (Pijls and De Bruyne, 1998; O’Brien and Nathan, 2008). The normal FFR was defined as 1 with FFR < 0.75 considered inadequate physiologic flow (Pijls and De Bruyne, 1998).

CTP data was analyzed using a dedicated neuroimaging software suite (MIStar; Apollo, Melbourne, Australia). In each set of CTP images, a region of interest (ROI) marker within the program was used to outline the carotid artery and internal jugular vein for arterial and venous references. The software analyzes the change in attenuation, measured in Hounsfield Units (HU), over time within the ROI and constructs an AIF and VOF curve with which CBF, CBV, and MTT were measured. The time to peak of AIF was determined by measuring the seconds it took to reach the highest AIF value in each animal.

The primary outcome of this study was CBF in normovolemic arrest animals compared to hypovolemic arrest animals undergoing OCM with an aortic cross clamp. Statistical analysis was performed using Stata v17.0 (Stat Corp LLC, College Station, TX, United States) and GraphPad Prism v8.0 (GraphPad Software Inc., San Diego, CA, United States). Mean values of CBV, CBF, and MTT were compared among normovolemic pre-arrest, normovolemic arrest, and hypovolemic arrest animals using multiple paired *t*-tests. Mean systolic pressures were also compared by paired *t*-tests. *p*-value less than 0.05 was considered statistically significant *a priori.* Raw study data can be made available upon request.

## Results

**FIGURE 2:**
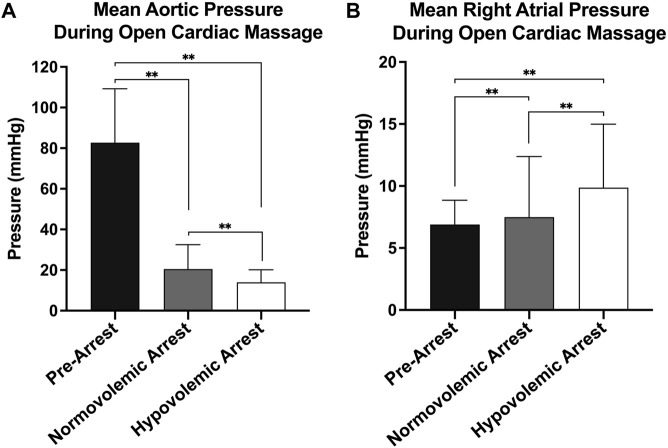
| Hemodynamics during open cardiac massage (OCM): **(A)** Mean aortic pressure with standard deviation of all three animal groups (pre-arrest, normovolemic arrest, and hypovolemic arrest), and **(B)** Mean right atrial (RA) pressure with standard deviation of all three animal groups. Statistical significance of p < 0.05 is denoted as * and p < 0.001 is denoted as **.

The mean weight of the animals enrolled was 42 ± 14 kg. The mean baseline aortic pressure (AoP) was 79 ± 12 mmHg in pre-arrest animals, 78 ± 15 mmHg in normovolemic arrest animals, and 80 ± 7 mmHg in hypovolemic arrest animals (Table 1). During OCM, the average AoP generated by compression was 21 ± 12 mmHg in normovolemic arrest animals and 14 ± 6 in hypovolemic arrest animals (*p* < 0.001). The RA pressure during normovolemic OCM was 8 ± 5 mmHg while the RA pressure during hypovolemic OCM was 10 ± 5 mmHg (*p* < 0.001; Figure 2).

**TABLE 1:** Baseline characteristics of all groups.

Variable, mean ± SD	Normovolemic pre-arrest	Normovolemic arrest	Hypovolemic arrest	*p*-value
Body mass (kg)	34.5 ± 5.1	57.8 ± 10.1	33.9 ± 6.2	0.03
Aortic pressure (mmHg)	78.6 ± 12.4	79.7 ± 7.0	78 ± 15	0.97
RA pressure (mmHg)	5.1 ± 3.2	6 ± 1.9	4 ± 2.7	0.71
CoPP (mmHg)	73.5 ± 2.2	73.6 ± 4.4	73.9 ± 9.3	0.99
Hemoglobin (g/dl)	11.4 ± 2.3	10.9 ± 0.9	11.5 ± 0.9	0.79
Hematocrit (%)	34.5 ± 7.5	30.5 ± 1.7	33.2 ± 2.4	0.41
Urea nitrogen (mg/dl)	9.6 ± 2.5	8.7 ± 1.5	10.8 ± 2.5	0.34
Glucose (mg/dl)	109.8 ± 44.8	97 ± 9.2	100.2 ± 39.2	0.83
Sodium (mmol/L)	138.5 ± 1.8	140 ± 3.6	138.7 ± 2.2	0.48
Potassium (mmol/L)	4.7 ± 0.6	4.1 ± 0.3	4.6 ± 0.5	0.13
Bicarbonate (mmol/L)	30.6 ± 2.1	31.2 ± 2.5	31.2 ± 2.1	0.91
Lactate (mmol/dl)	2.6 ± 0.6	2.7 ± 0.7	2.3 ± 0.5	0.44

SD, standard deviation; RA, right atrial; CoPP, coronary perfusion pressure.

**FIGURE 3:**
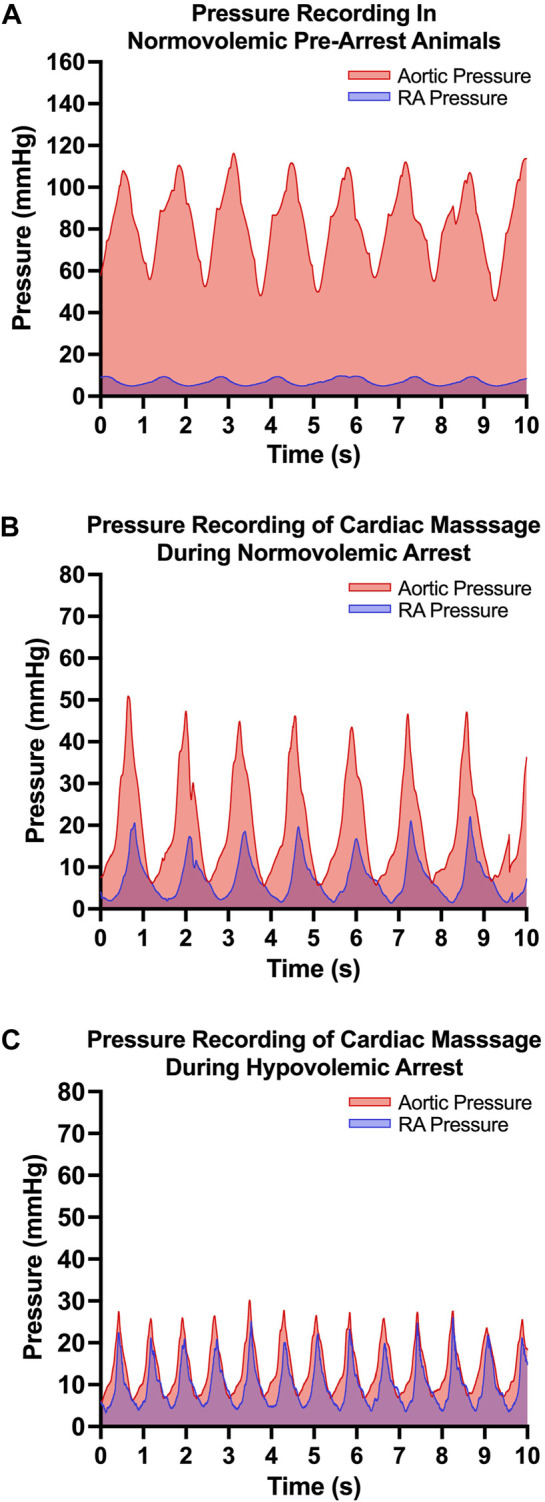
| Open cardiac massage (OCM) performance metrics: **(A)** Representative pressure tracing of aortic and right atrial (RA) pressure in the normovolemic pre-arrest model, **(B)** Representative pressure tracing of aortic and right atrial (RA) pressure during OCM in a normovolemic arrest model and **(C)** Representative pressure tracing of aortic and RA pressure during OCM in a hypovolemic arrest model.

**FIGURE 4:**
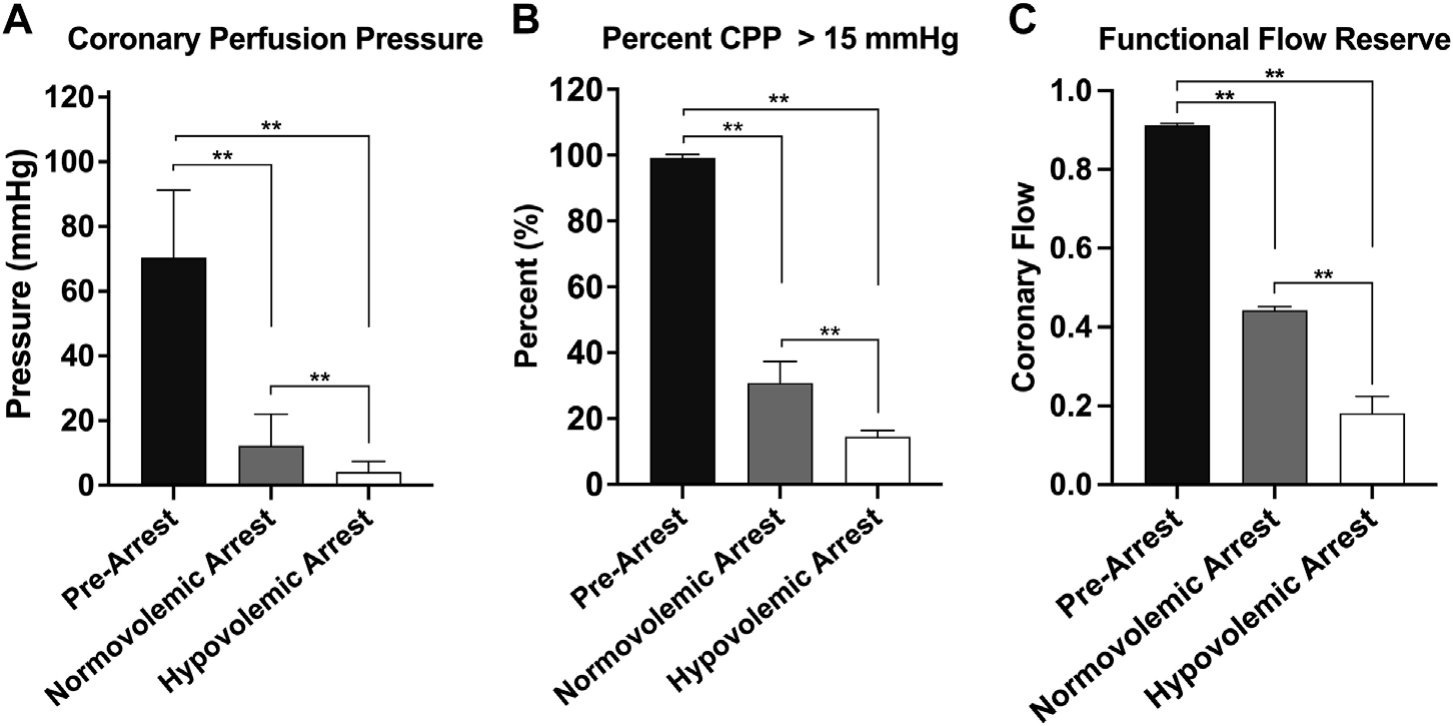
| Graphical representations of the three cohorts during open cardiac massage: normovolemic pre-arrest, normovolemic arrest, and hypovolemic arrest. **(A)** Mean cerebral perfusion pressure (CoPP), **(B)** the percentage of pressure tracing segments where CoPP > 15 mmHg, and **(C)** the functional flow reserve. Statistical significance denoted on graph. Statistical significance of p < 0.05 is denoted as * and p < 0.001 is denoted as **.

The efficacy of OCM performed on the normovolemic versus hypovolemic arrested animals was assessed (Figure 3). The average CoPP in normovolemic and hypovolemic arrest animals was 12 ± 10 mmHg and 4 ± 3 mmHg respectively (*p* < 0.001). The peak CoPP was 39 mmHg in normovolemic arrest animals and 12 mmHg in hypovolemic arrest animals (*p* < 0.001). When discreet 10 s segments were analyzed during the time OCM was performed, the average percentage of segments achieving a CoPP > 15 mmHg was 31% in normovolemic arrest and 15% in hypovolemic arrest (*p* < 0.001; Figure 4). When coronary flow was assessed, the FFR was significantly lower during cardiac arrest in both normovolemic and hypovolemic animals compared to the pre-arrest animals (*p* < 0.001; Figure 4C). The hypovolemic arrest animals had a lower FFR compared to normovolemic arrest animals (0.4 ± 0.02 vs. 0.2 ± 0.1; *p* = 0.003). However, both cardiac arrest groups were well below the < 0.75 FFR definition that signifies inadequate flow. Table 2.

**TABLE 2:** Hemodynamic characteristics of normovolemic arrest and hypovolemic arrest during open cardiac massage.

Variable, mean ± SD	Normovolemic pre-arrest	Normovolemic arrest	Hypovolemic arrest	Pre-arrest vs. normovolemic arrest	Pre-arrest vs. hypovolemic arrest	Normovolemic arrest vs. hypovolemic arrest
Aortic pressure (mmHg)	82.7 ± 16.5	20.5 ± 12.1	14.0 ± 6.1	<0.001	<0.001	<0.001
RA pressure (mmHg)	5.9 ± 1.9	7.5 ± 4.9	9.9 ± 5.1	<0.001	<0.001	<0.001
CoPP (mmHg)	70.4 ± 20.8	12.3 ± 9.7	4.1 ± 3.3	<0.001	<0.001	<0.001
CoPP > 15 mmHg (%)	99.3 ± 0.9	30.9 ± 6.5	14.5 ± 1.9	0.002	<0.001	0.04
Coronary FFR	0.9 ± 0.01	0.4 ± 0.02	0.2 ± 0.1	<0.001	<0.001	0.003
CBV (ml/100 g)	4.6 ± 1.5	4.6 ± 2.9	0.5 ± 0.4	0.97	0.002	0.03
CBF (ml/min/100 g)	51.9 ± 12.1	7.5 ± 8.1	4.9 ± 6.0	0.003	0.003	0.58
MTT (s)	3.2 ± 1.5	3.2 ± 2.2	3.5 ± 1.3	0.99	0.76	0.8
AIF (Hu)	35.8 ± 66.3	6.1 ± 6.1	4.5 ± 10.4	0.007	<0.001	0.001
VOF (Hu)	11.1 ± 7.2	6.8 ± 5.8	1.5 ± 1.8	<0.001	<0.001	<0.001

SD, standard deviation; RA, right atrial; CoPP, coronary perfusion pressure; FFR, functional flow reserve; CBV, cerebral blood volume; CBF, cerebral blood flow; MTT, mean transit time; AIF, arterial input function; HU, hounsfield units; VOF, venous output function; AIF, and VOF, were calculated as the change in contrast concentration (HU) in cerebral tissue over time.

The mean AIF and VOF curves of all included animals at each second of contrast transit were graphed with the 95% confidence intervals for baseline, normovolemic arrest, and hypovolemic arrest cohorts in Figures 6A–C. The arterial and venous HU peaks were similar between normovolemic arrest and hypovolemic arrest study groups; however, the time to peak elongated from spontaneous circulation to normovolemia to hypovolemia (6 ± 0.4 vs. 16 ± 4 vs. 20 ± 7 s; *p* = 0.007).

**FIGURE 5:**
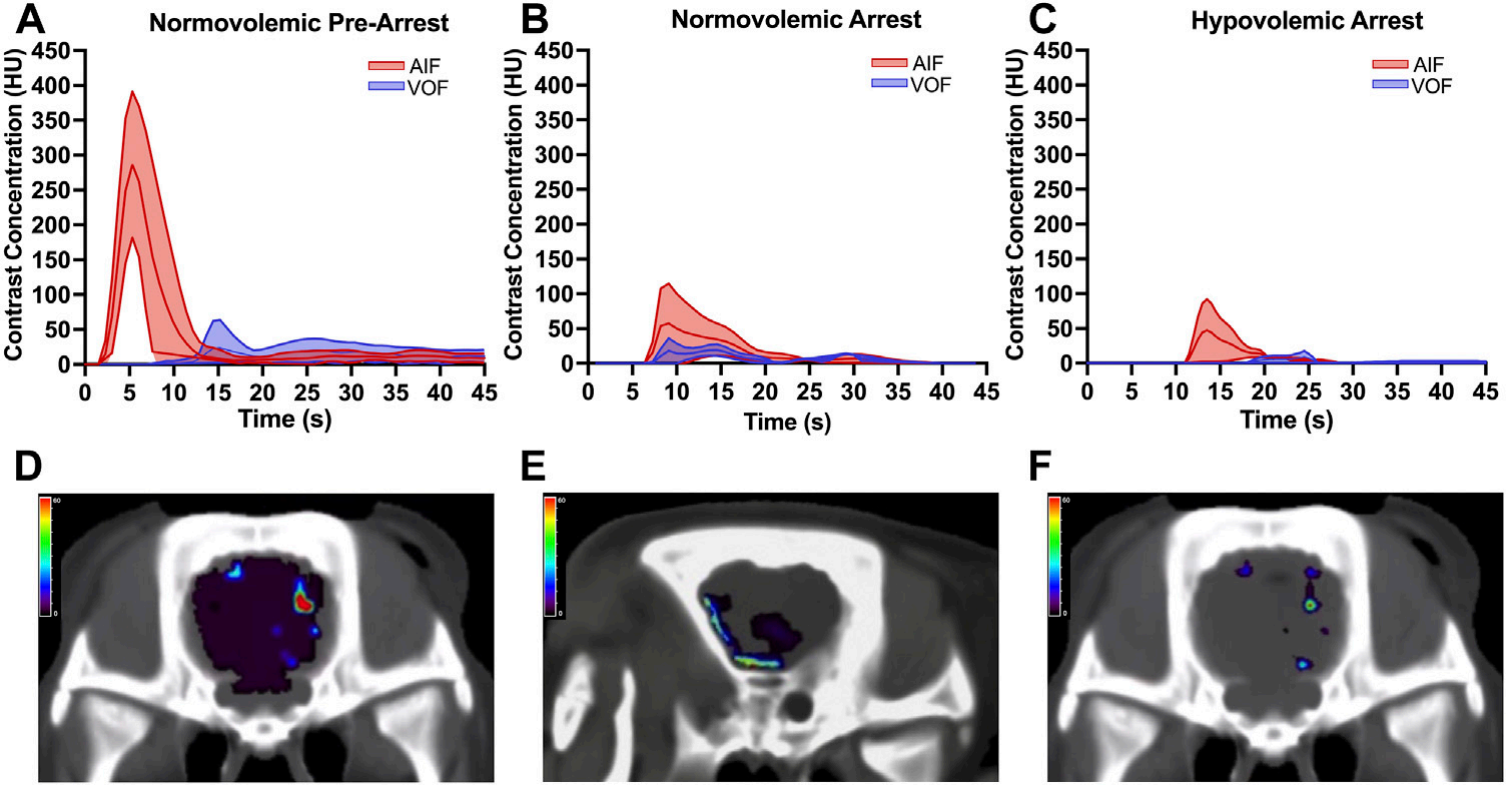
| Arterial input function (AIF) and venous output function (VOF) curves derived from change in cerebral tissue contrast concentration over time measured in Hounsfield units (HU) using the standardized region-of-interest tool for the internal carotid artery and internal jugular vein for **(A)** normovolemic pre-arrest, **(B)** normovolemic arrest, and **(C)** hypovolemic arrest. Data was collected from all animals in each group. The central line graphed over time represents the mean and the shaded area represents the 95% confidence interval. The arrest animals underwent computed tomography acquisition following 5 min of open cardiac massage (OCM). Corresponding heatmaps demonstrate the cerebral blood flow (CBF) during OCM, which is measured by the slope of the AIF, for **(D)** normovolemic pre-arrest, **(E)** normovolemic arrest, and **(F)** hypovolemic arrest. The heat map legend represents the CBF measured in ml/min/100 g using red to represent the highest CBF and purple to represent the lowest CBF.

**FIGURE 6:**
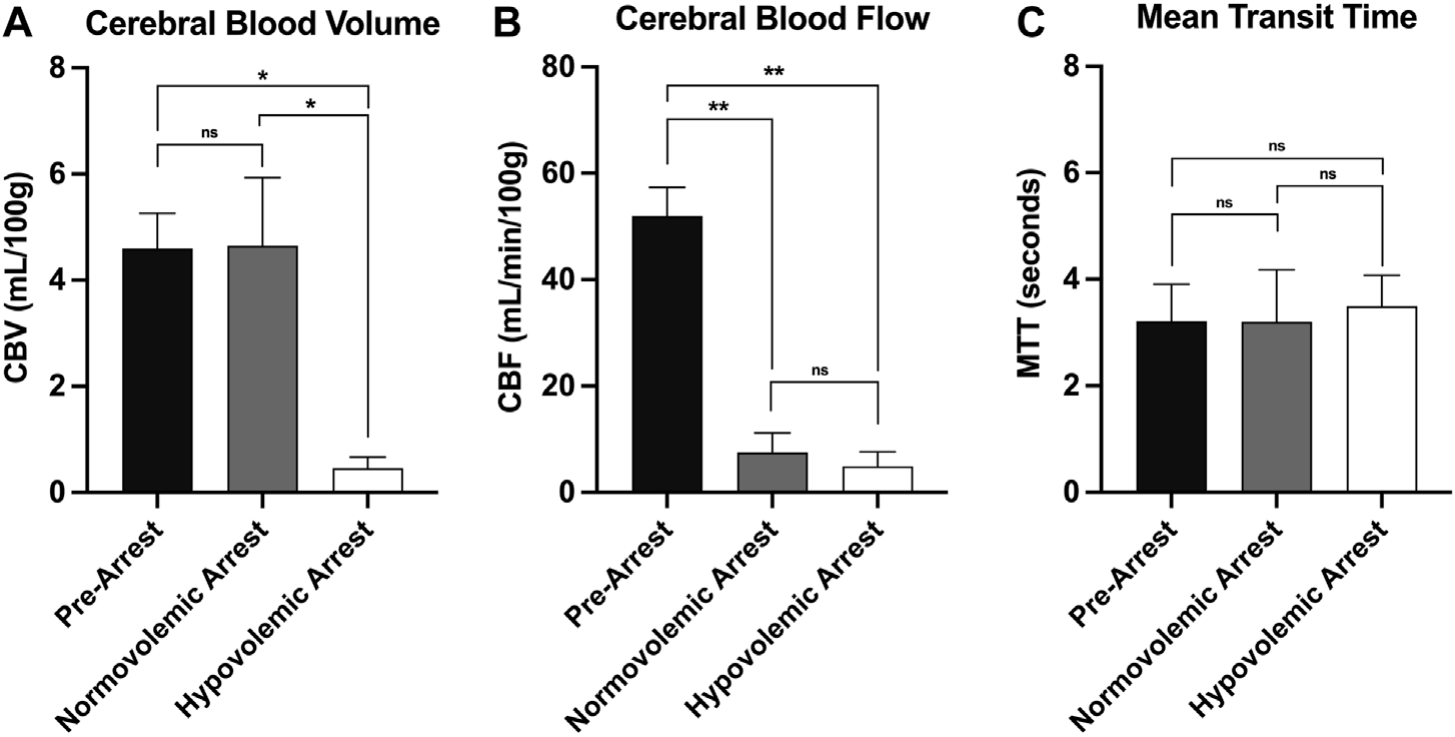
| CT perfusion metrics of cerebral perfusion with comparison of **(A)** cerebral blood volume, **(B)** cerebral blood flow, and **(C)** mean transit time during normovolemic pre-arrest, normovolemic arrest, and hypovolemic arrest with statistical significance denoted on graph. Statistical significance of p < 0.05 is denoted as *, p < 0.001 is denoted as **, and non-significant p-values are denoted as ns.

The mean CBV during normovolemia was 5 ± 2 ml/100 g compared to 0.5 ± 0.4 ml/100 g during hypovolemia OCM (*p* = 0.02). CBV was reduced in the hypovolemic arrest animals compared to normovolemic arrest (5 ± 3 vs. 0.5 ± 0.4 ml/100 g; *p* = 0.005), while MTT remained similar (3 ± 2 vs. 4 ± 1 s; *p* = 0.95) (Figure 5). CBF was reduced in both normovolemic arrest and hypovolemic arrest compared to pre-arrest animals (8 ± 8 vs. 5 ± 6 vs. 52 ± 12 ml/min/100 g; *p* < 0.001). Corresponding CTP heat maps in Figure 6 are three examples of the cohort-specific changes in CBF, which is measured by the slope of the AIF, during OCM.

## Discussion

Traumatic exsanguination cardiac arrest is a catastrophic and challenging clinic scenario in the best of circumstances. Even when ROSC is achieved, poor neurological outcomes are persistently poor and long-term outcomes are dismal (Gräsner et al., 2011; Duchateau et al., 2017; Edwards et al., 2022b). Up to half of these patients die within 6 months of discharge and about half of surviving patients have debilitating functional impairment and require around-the-clock care. These motivate the need for new surgical and critical care solutions to traumatic arrest that facilitate cardiopulmonary resuscitation while preserving the central nervous system (CNS). An understanding of brain perfusion during traumatic arrest, and RT and OCM are critical to these ends.

This study demonstrates that CTP is useful for actually evaluating techniques that augment cerebral perfusion in the setting of both hypovolemic and normovolemic arrest. The results found conventional OCM during RT produces suboptimal global cerebral perfusion regardless of blood volume status. The vast difference between CBV, CBF, and SBP during normovolemia baseline and animals receiving OCM demonstrates the severe shortcoming of OCM as an attempt to artificially replicate spontaneous circulation and perfusion.

Cerebral perfusion pressure (CPP) was not directly measured in this study as it is calculated by subtracting the intracranial pressure (ICP) from the MAP and ICP was not obtained in this study (Fantini et al., 2016). In the setting of negligible ICP (i.e., remaining at a constant baseline between 5–15 mmHg) the CPP can be estimated based on MAP alone (Pinto et al., 2007; Fantini et al., 2016). As the animals in this study did not have undergo any traumatic injuries to the brain, the CPP was likely significantly decreased in both the normovolemic and hypovolemic animals. Additionally, CBF is linearly correlated with CPP, which acts as the driving pressure for CBF (Schumann et al., 1998; Fantini et al., 2016; Kameyama et al., 2022). The CBF was significantly decreased during cardiac arrest in both normovolemic and hypovolemic animals which further validates the estimated changes in CPP based on MAP.

The similar MTT between the groups is accounted for by slower overall flow velocity coupled with lower blood volume indicating that a given region of brain tissue is perfused by less volume of blood, for the same amount of time as normal physiologic blood flow. This effectively reduces oxygen delivery to the cerebrum when it is needed most. This shortcoming is likely a significant contributor to neurologic outcomes in trauma patients that present in cardiac arrest. Even when performed by experienced operators, OCM generated poor SBP and thus poor cerebral perfusion when compared to spontaneous, coordinated cardiac contractions.

In addition to anoxia and ischemia, various mechanisms have been proposed to explain poor neurologic outcomes such as reperfusion injury, electrolyte, and pH derangements. Crucially, timing and quality of CPR has been shown to be a strong predictor of neurologic outcome (Nielsen et al., 2013; Hughes and Perkins, 2020). The goal of cross-clamping and compressions is to perfuse the heart and CNS, and we have previously shown that maintaining a coronary perfusion pressure (CoPP) above 15 mmHg is crucial to perfusing the myocardium adequately and achieving ROSC (Lurie et al., 2016; Madurska et al., 2021); however, generating and maintaining adequate CoPP *via* OCM is difficult to achieve. Normovolemic animals in arrest receiving OCM were more likely to reach adequate levels of CoPP compared to hypovolemic animals in arrest.

Given that OCM generates poor coronary perfusion primarily in hypovolemic animals, it is not surprising that cerebral perfusion suffers as well. However, normovolemic animals in arrest did have adequate CoPP during OCM, yet cerebral perfusion was as equally poor as hypovolemic arrest animals. This study represents the first-time cerebral perfusion data is collected during RT and OCM *in vivo* and provides a physiologically relevant model for assessing cerebral perfusion during normovolemic and hypovolemic arrest. Furthermore, in general, the CTP perfusion protocol utilized in this study affords a non-invasive method of assessing global cerebral perfusion in a swine model.

Several clinical methods are used to improve neurological outcomes in patients presenting in traumatic arrest. Earlier intervention in the form of prehospital thoracotomy has been studied in specific instances of tamponade in certain regions (Paradis et al., 1990). Therapeutic hypothermia has also been shown to afford nearly two-fold benefit for favorable neurological outcomes measured by the cerebral performance category scoring system (Stiell et al., 2009; Davies and Lockey, 2011; Arrich et al., 2016). “Good cerebral performance” is primarily defined as conscious, alert, capable of normal life with or without minor neurological or psychological deficits. (Stiell et al., 2009).

This study highlights the need for improved and rapid resuscitative techniques for patients in exsanguination cardiac arrest. Some experimental systems such as selective aortic arch perfusion and extracorporeal CPR have been explored which show improved overall coronary perfusion (Madurska et al., 2020; Madurska et al., 2021). Further studies are needed to test systems like these for their effect on cerebral perfusion as well. A “holy-grail” solution to traumatic cardiac arrest would have a high rate of cardiopulmonary salvage, while also preserving the CNS. With the combination of improved coronary and cerebral perfusion during resuscitation using these novel techniques, it is possible that neurological outcomes can be dramatically improved.

This study has important limitations. First, this study is a small sample size of swine. Secondly, while we arbitrarily chose the gender of the animals, the included animals were male without any female swine included. This may decrease the generalizability of our findings as it focuses on the physiology of male swine and future work should include female animal models (Zucker and Beery, 2010). Additionally, swine are an ideal system due to their cardiovascular, physiologic, and neurologic similarity to humans, but they are not identical. It is also impossible to replicate the myriad circumstances, injury patterns, and physiologic derangement across the spectrum of bleeding trauma patients. The animals on this protocol, however, are a common physiologic and otherwise similar representation of a salvageable exsanguinated trauma patient, and the sample size was predetermined by power analysis. Here, we use a validated CT perfusion protocol to assess cerebral perfusion and flow. This does provide information about broader cerebral ischemia, however, does not necessarily tell us about neural injury and provides no information on cellular metabolic function or the reversibility thereof. As such, metabolic and molecular physiologic changes during OCM and associated ROSC outcomes were not studied and should be investigated in future research. Other future studies may compare other arms of intervention for severe hypovolemia, including selective aortic perfusion, other mechanisms of aortic occlusion, or vasoactive medications.

## Conclusion

Resuscitative thoracotomy and cardiac massage result in poor systolic blood pressure and achieve inadequate cerebral perfusion, likely contributing to poor neurologic outcomes. This study is the first demonstrating cerebral perfusion during RT and OCM on a representative model of cardiopulmonary and neurological systems. RT with OCM is a poor technique to achieve adequate cerebral perfusion regardless of blood volume status and novel resuscitative techniques need to be investigated that specifically target cerebral perfusion to improve outcomes.

## Data Availability

The raw data supporting the conclusion of this article will be made available by the authors, without undue reservation.
